# Supplementary figures and images for: Capsules of the diatom Achnanthidium minutissimum arise from fibrillar precursors and foster attachment of bacteria
Source: PeerJ. 2015 Mar 26;3:e858. doi: 10.7717/peerj.858 (PMC4380156; doi:10.7717/peerj.858)

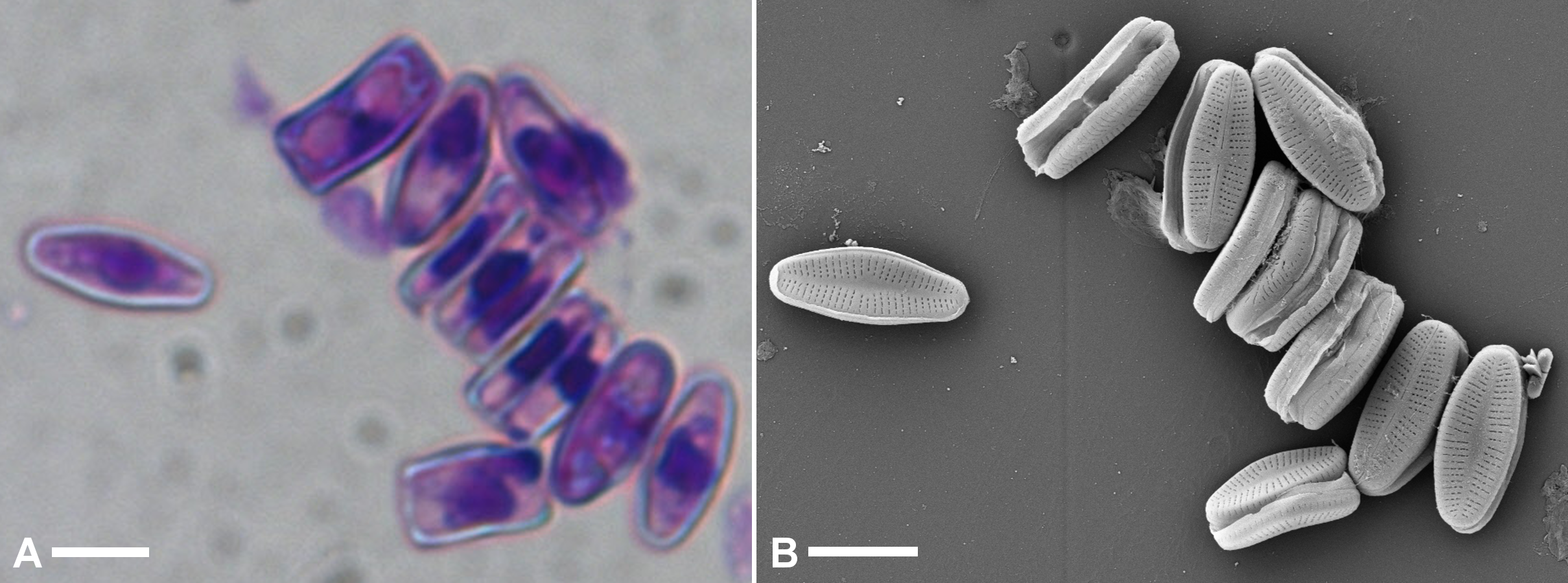

Supplement: Figure S1 — Demonstration of the same technique used to identify the appearance of xenic biofilms and dehydrated capsule material in SEM (main Fig. 2) in axenic cultures after 31 days of incubation with much fewer adherent cells and no capsules. [file peerj-03-858-s001.png]

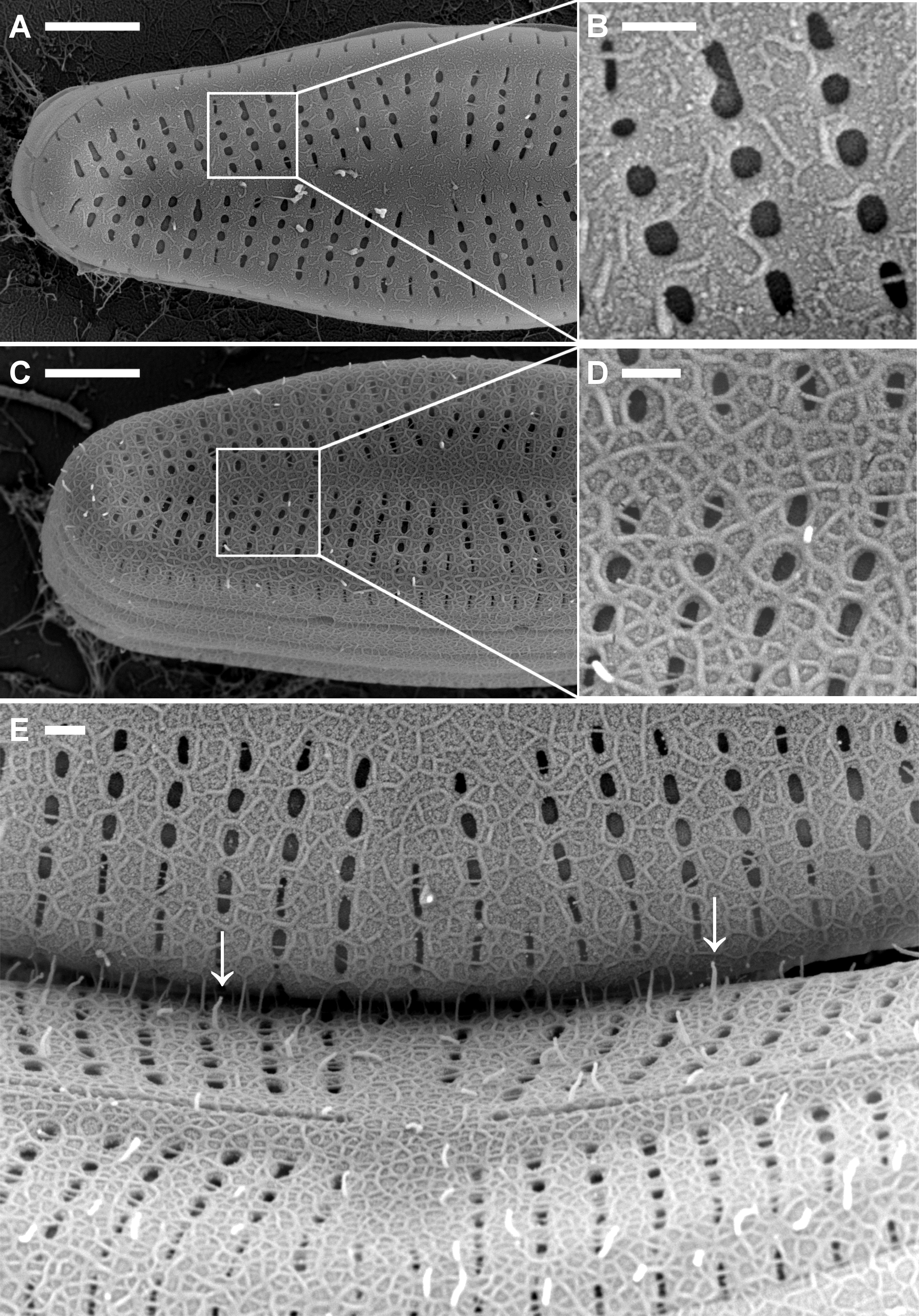

Supplement: Figure S2 — Samples were prepared for SEM after 20 days of incubation. (A) (scale bar: 1 µm) & (B) (scale bar: 200 nm): Frustules with few, short fibrils, which were not found in xenic biofilms. C (scale bar: 1 µm) & (D) (scale bar: 200 nm): Frustule with medium-dense fibrillar mesh, as also seen in xenic biofilm (main Fig. 4A). (E) (scale bar: 200 nm): Fibrils are not only flatly attached to the frustule but also stick out into space and make contact with other cells (arrows), as also seen in xenic cultures (main Fig. 4B). [file peerj-03-858-s002.png]
